# Assessment of Noise Levels of Equipment Used in the Practical Dental Teaching Activities

**DOI:** 10.1155/2021/6642560

**Published:** 2021-03-01

**Authors:** Meriem Amine, Zineb Aljalil, Asmaa Redwane, Ikram Delfag, Imane Lahby, Anas Bennani

**Affiliations:** ^1^Fixed Prosthesis Department, Faculty of Dentistry of Casablanca, Hassan II University of Casablanca, Casablanca, Morocco; ^2^Epidemiology Laboratory, Faculty of Dentistry of Casablanca, Hassan II University of Casablanca, Casablanca, Morocco; ^3^Faculty of Dentistry of Casablanca, Hassan II University of Casablanca, Casablanca, Morocco

## Abstract

**Introduction:**

Practical activities in dentistry are characterized by a high noise level that can have adverse effects on the hearing health of professors, students, and teaching staff. The objective of our study was to make an assessment of the noise level during the practical fixed prosthodontics activities in the Faculty of Dentistry of Casablanca.

**Materials and Methods:**

We conducted a descriptive cross-sectional study to measure the noise level in the practical room of fixed prosthodontics. The measurements were obtained during 4 sessions over a duration of 2 hours and 30 minutes, each with the use of a SdB + sound level meter at 4 different locations.

**Results:**

The results showed the following: an average value of 69.35 dB (*A*) for the first practical session (south), an average value of 71.07 dB (*A*) for the 2nd practical session (east), an average value of 70.36 dB (*A*) for the 3rd practical session (west), and an average value of 72.06 dB (*A*) for the 4th practical session (center of the room). *Discussion and Conclusion*. The results obtained are similar to the results found in previous studies in other countries. These results are below the thresholds of the legislation and international standards. However, we have recorded punctual peaks that exceed the recommended level, requiring the introduction of the means of prevention and the measures of safety against the noise as well at the level of the practical activity classroom and the realization of more in-depth studies concerning the evaluation of the daily exposure of the professors, students, and teaching staff to noise.

## 1. Introduction

Dental practitioners are exposed to risks of all kinds that include infectious and communicable diseases, musculoskeletal pathologies, ionizing radiation, and also long exposure to high sound levels following the use of rotary instruments that may cause adverse effects and, in some cases, may lead to hearing loss [1]. These effects can be expressed both at the atrial level and at the extra-auricular level [2, 3] (sleep disorders, cardiovascular disorders, stress, etc.).

The acoustic environment of educational and teaching activities in a faculty of dentistry is characterized by high noise levels compared to other areas of education, as they require the use of various equipment that emit noise by many students at the same time [4].

International standards for noise at work set the exposure level at 80 dB for 8 working hours [5]. These standards predict, in part, the design performance of teaching premises, which must be built with specific materials to reduce noise [5].

The practical classroom of fixed prosthodontics is equipped with about 40 workstations, each occupied by a student and work at the same time, and these students are supervised by a team of professors.

Taking all these factors into account, one question arises: is the noise level during practical activities of fixed prosthesis part of the standards?

In order to answer this question, we proposed to conduct a descriptive cross-sectional study to evaluate the level of noise in the practical classroom of fixed prosthesis during sessions of practical activities in the Faculty of Dentistry of Casablanca.

## 2. Materials and Methods

### 2.1. Description of the Local

The practical activity room with an area of 153 m^2^, built with ordinary materials without soundproofing, is located on the 2nd floor, away from the streets and major avenues of Casablanca. It consists of a special training room reserved for carrying out the practical activities of the students, a plaster room, and a technician's room.

### 2.2. Local Content

The workstations occupy 32 m^2^ of the surface of the room, equivalent to 25.52% of the entire room. The posts are in the form of four benches arranged to occupy all sides of the room. The room also contains, in addition to the benches, a desk reserved for the supervisors.

### 2.3. Sources of Noise in the Practical Activity Room

#### 2.3.1. Outdoor Noises

These include noises coming from outside the practical room of fixed prosthodontics.

#### 2.3.2. Inner Sounds

During each practical session lasting 2.5 hours, each of the 39 students uses a rotating material of their own. The supervisors remain the same for a period of 5 hours (half-day), while students change between sessions of practical activities.

The themes of the practical activities are divided according to a program where the use of rotating equipment is essential for each session of practical activities. The theme of the different sessions of our study is the same and consists of preparing metal-ceramic crowns on a Frasaco tooth (resin) using a diamond burr mounted on a contra-angle. The rotating parts, in operation, cannot be adjusted in power and are therefore always at maximum power.

### 2.4. Materials Measurement

The measurement equipment used for this study is a calibrated SdB + sound level meter that operates in the classical mode for measuring the sound pressure level (Lp) and the maximum sound pressure level (Lpmax).

The sound level meter is placed at a height of 1.20 meters above the ground, at the same level as the ear, to simulate the intensity of the noise reaching the eardrum, towards the noise source. The measuring range is 30 to 110 dB. The selected measurement interval is 30 seconds.

Since the sound level meter did not have a recorder, we opted for the choice of a high-precision camera type, GoPro Hero 3+ Black Edition, programmed to take pictures every 30 seconds which corresponds to our measured interval.

The background noise was measured for 10 minutes while the room is empty, respecting the same conditions of measurement of the practical dental teaching activities.

The study was conducted over a duration of 4 sessions which means 10 hours of measurement, under the same working conditions. We evaluated the noise level in 4 separate points in the practical activity classroom. One point is located in the center of the practical classroom, and each of the other three points is located near an outside noise source, namely east, west, and south.

## 3. Results

The results evaluated with a sound level meter are presented in the form of tables and graphs.

**Figure 1:**
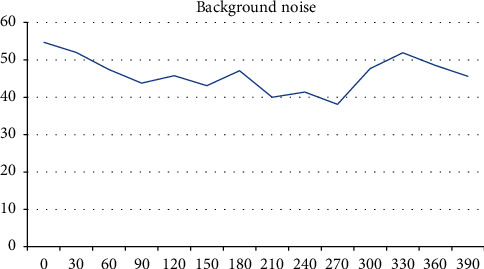
Background noise.

During the measurement of the background noise, we noted a sound level ranging from 38.1 dB (*A*) to 54.7 dB (*A*), with an average value of 45.01 dB (*A*) and a standard deviation of 4.45 dB (*A*) (Figure 1).

**Figure 2:**
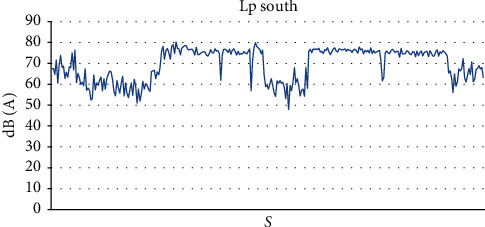
Sound level Lp south of the practical classroom.

In the south of the practical activity classroom, we noted a sound level ranging from 47.9 dB (*A*) to 80.2 dB (*A*), with an average value of 69.35 dB (*A*) and a standard deviation of 7.82 dB (*A*) (Figure 2) and a maximum sound level ranging from 78.9 dB (*A*) to 85.7 dB (*A*) with an average value of 82.96 dB (*A*) (Table 1).

**Figure 3:**
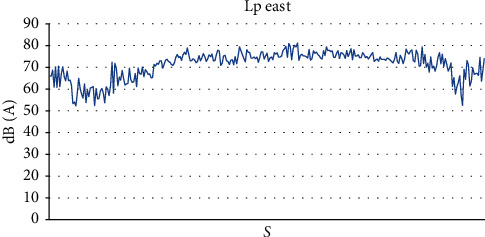
Sound level Lp east of the practical classroom.

In the second phase, we found a noise level in the east of the practical activity classroom, from 52.3 dB (*A*) to 81.1 dB (*A*), with an average value of 71.07 dB (*A*) and a standard deviation of 6.3 dB (*A*) (Figure 3) and a maximum noise level between 77.3 dB (*A*) and 86.1 dB (*A*) with an average value of 83.2 dB (*A*) (Table 2).

**Figure 4:**
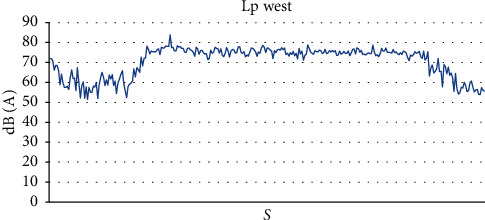
Sound level Lp west of the practical classroom.

In the west of the practical classroom, we noted a sound level ranging from 51.5 dB (*A*) to 83.9 dB (*A*), with an average value of 70.3 dB (*A*) and a standard deviation of 7.6 dB (*A*) (Figure 4) and the maximum sound level varies between 63.8 dB (*A*) and 86.3 dB (*A*) with an average value of 82.96 dB (*A*) (Table 3).

**Figure 5:**
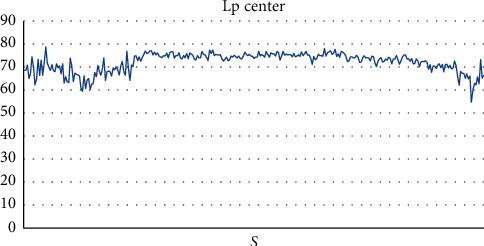
Sound level Lp in the center of the practical classroom.

At the center of the practical classroom, we noted a sound level ranging from 54.7 dB (*A*) to 78.7 dB (*A*), with an average value of 72.06 dB (*A*) and a standard deviation of 4.15 dB (*A*) (Figure 5) and a maximum sound level ranging from 87.7 dB (*A*) to 87.8 dB (*A*) with a mean value of 87.8 dB (*A*). (Table 4).

We note a statistically significant difference (*p* < 10^−6^) between the mean values of the 4 sessions of practical activities.

The comparison of the average sound level recorded at the center of the room with those recorded at the other locations shows that the center was noisy and the observed difference here is statistically significant (*p* < 10^−6^).

## 4. Discussion

### 4.1. Background Noise

Numerous studies have measured environmental noise level [6–9] over a period of 10 minutes [10]. We know the environmental noise level depends on the number of operators, the time of the day, noise from outside through open windows (crowded streets and traffic), and finally radio and television [6, 9].

According to these studies, the maximum values vary between 40 dB (*A*) [10], 43 dB (*A*) [9], 55.0 dB (*A*) [6], and 65 dB (*A*) [8]. In our study, the maximum noise level was 54.7 dB (*A*), and we note the presence of construction work nearby at the time of measurement.

For a 401 m^s^ volume room like ours, an average background noise of 45.01 dB (*A*) exceeds the level recommended by the National Research Council of Canada.

To determine whether background noise influences total noise measurements, corrective factors are statistically established [11].

In the study presented, the difference between the level of the total noise and that of the background noise is greater than 10 dB and the amount to be added to the total noise level is zero. In such cases, no adjustment factor is needed [11].

### 4.2. Noise Distribution during Practical Sessions

For the last four sessions of the practical activities, we note that the noise level remains low during the first 30 min approximately. This is explained by the time devoted to the presentation of recommendations to be followed by the students, unlike the first session being realized at the beginning of the vacation. As soon as the students begin to prepare their teeth, the noise level increases for about 2 h15 min of the practical session. The noise level drops to the initial low level at the end of the practical session while the students return their equipment and free the practical activity classroom. The sound level can reach 80.2 dB (*A*) only once the session begins.

### 4.3. Homogeneity of Noise

We evaluated the noise level in 4 separate points in the practical activity classroom. One point is located in the center of the classroom, and each of the other three points is located near a source of external noise. This choice was intended to study the homogeneity of the noise in the room and the influence of the outside noise.

When comparing the averages of the four sessions of practical activities, we found a statistically significant difference (*p* < 0.05). This means that the noise is not evenly distributed in the practical classroom, and there are noisier places than others.

In order to have a representative value allowing comparison of our results with the other studies, we chose the average of the center of the practice classroom which is 72.65 dB (*A*) since the center is noisier than the other points of the room.

### 4.4. Comparison of Sound Level with the Literature

The results of this study are comparable to the results of other international studies on noise in dental settings.

In their study, Singh et al. showed a mean value of 80.1 dB (*A*) in the center of the classroom while 70 contra-angles were working at the same time [12]. Fernandes et al. showed an average value of 75.2 dB (*A*) in the Faculty of Dentistry of Porto [13]. They evaluated the preclinical noise level emitted by the contra-angles working on the acrylic resin. In comparison with this value, our average (72.65 dB (*A*)) remains lower. This can be explained by the number of contra-angles used; in our study, only 39 contra-angles worked at the same time.

In addition, other studies found values slightly lower than our study. Bahannan et al. in 1993 evaluated clinically the sound level emitted by new and good-working contra-angles, working on a natural extracted tooth using several types of milling cutters (diamond mill, tungsten carbide, and steel), and found an average value (69.71 dB (*A*)) lower than our study (72.65 dB (*A*)) knowing that the working conditions are not exactly the same [14].

Similarly, studies by Parkar et al. [15] and Sushi et al. [4], working with contra-angles on acrylic resins, found average values of 69.28 dB (*A*) and 69.3 dB (*A*), respectively. In comparison with all these studies, our results are close to the literature.

According to the National Research and Safety Institute, for an 8-hour workday, hearing is considered to be in danger from 80 dB (*A*) [16]. Indeed, the higher the acoustic level, the more the necessity to limit the exposure time.

Referring to the international standards that govern the level of worker exposure to noise during work, the average of our study is well below the statutory limits. However, despite this, we cannot confirm the absence of risk for hearing.

### 4.5. Maximum Values Recorded

During the 5 measurement sessions, we recorded noise level peaks represented by Lpmax values.

We noted, for the 1st practical session, a maximum value of 85.7 dB (*A*) and for the 2nd session, 86.31 dB (*A*). For the 4th session, we noted a maximum value of 86.3 dB (*A*).

All these values are well above the action thresholds of the legislation and the level recommended by the international standards for noise exposure at work.

By having Lpmax values that exceed 85 dB (*A*), this confirms the presence of risk of noise exposure for teachers and educational staff, in particular, and students.

Exposure of workers to high levels of noise can cause two health effects, auditory and extra-auditory [2, 3].

The effects of noise are difficult to quantify because the tolerance levels between the populations are different and the types of noise vary considerably [17].

## 5. Conclusion

The acoustic environment of practical activities is characterized by a high level of noise generated by various rotating materials handled by students that can put them and professors and educational staff at risk.

Therefore, it seemed necessary to evaluate noise levels in such environments in a faculty of dentistry. The purpose of this study was to respond to this need.

The results of our study indicate an average sound level that does not exceed the thresholds recommended by law and international standards.

However, we recorded punctually higher values than the allowable level, thus putting in risk the hearing of the set of supervising staff.

In this context, it would be interesting to evaluate the daily dose of noise exposure for students, professors, and teaching staff using another measuring device, exposure meter or dosimeter, and to determine the long-term effects of noise on these individuals through work based on audiometric testing and questionnaires.

This study must therefore enable us to continue work in this direction.

## Figures and Tables

**Table 1:** Maximum sound level Lpmax south of the practical room.

Lpmax dB(A)	*N*	%
78.9	23	7.7
80.4	2	0.7
80.9	1	0.3
81.1	54	18.1
81.8	12	4.0
81.9	44	14.7
82.1	51	17.1
85.1	1	0.3
85.7	111	37.1
Total	299	100

**Table 2:** Maximum sound level Lpmax east of the practical classroom.

Lpmax	*N*	%
77.3	49	16.4
77.5	1	0.3
80.3	3	1.0
81.7	42	14.0
82.2	24	8.0
82.7	15	5.0
83.9	2	0.7
85.4	108	36.1
86.1	55	18.4
Total	299	100

**Table 3:** Maximum sound level Lpmax west of the practical classroom.

Lpmax	*N*	%
63.8	22	7.4
67.9	2	0.7
68.2	5	1.7
72.0	3	1.0
72.5	2	0.7
73.9	1	0.3
75.5	2	0.7
75.7	2	0.7
78.5	23	7.7
80.7	5	1.7
82.0	6	2.0
84.8	10	3.3
86.3	216	72.2
Total	299	100

**Table 4:** Maximum sound level Lpmax in the center of the practical classroom.

Lpmax	*N*	%
87.7	1	0.3
87.8	298	99.7
Total	299	100

## Data Availability

The data are conserved in the fixed prosthesis department of the Faculty of Dentistry of Casablanca.
